# Spatio-temporal characterization of drought variability in data-scarce regions using global precipitation data: a case study in Cauto river basin, Cuba

**DOI:** 10.1038/s41598-024-61709-9

**Published:** 2024-05-22

**Authors:** Anh Phuong Tran, Bao Chung Tran, Siliennis Blanco Campbell, Nam Anh Nguyen, Dieu Hang Tran, Thanh Thuy Nguyen, Anh Duc Nguyen, Hong Son Duong

**Affiliations:** 1 Water Resources Institute, No. 8 Phao Dai Lang, Dong Da, Hanoi, Vietnam; 2 Granma Provincial Delegation of Hydraulics Resources, Amdo Estévez s/n, Bayamo, Granma Cuba; 3 Thuyloi University, 175 Tay Son, Dong Da, Hanoi, Vietnam

**Keywords:** CHIRPS, Standardized precipitation index (SPI), Thiessen polygon, Drought characteristics, Trend analysis, Spatial analysis, Climate sciences, Hydrology

## Abstract

Drought is considered the most severe water-related disaster in the Cauto river basin, which is the longest river and the main agricultural producer in Cuba. Better understanding of drought characteristics is crucial to drought management. Given the sparsity of ground-based precipitation observations in the Cauto, this study aims at using gridded global precipitation to analyze the spatio-temporal variations of drought in this river basin. Firstly, the monthly Climate Hazards Group InfraRed Precipitation with Station data (CHIRPS) was calibrated with the gauged precipitation using the Thiessen polygon-based method and linear least squares regression equations. Then, the gridded standardized precipitation index (SPI) with time scales of 3, 6, 9 months and drought characteristics, namely, drought frequency, duration and intensity were calculated using the calibrated CHIRPS. Finally, the spatio-temporal analysis was performed to investigate the variations of drought in the Cauto river basin in time and space. The obtained results show that the calibrated CHIRPS is highly consistent with the gauged observations and is capable of determining the magnitude, time, and spatial extent of drought events in the Cauto river basin. The trend analysis by the Mann–Kendall test reveals that although the trend is not statistically significant, the SPI tends to decrease with time in the dry season, which indicates the more severe drought. The spatial analysis indicates that the lower altitude area of the Cauto river basin is suffered from longer drought duration and higher drought intensity than the upper one. This study expresses the importance of open global precipitation data sources in monitoring and quantifying drought characteristics in data-scarce regions.

## Introduction

Drought is a prolonged dry period in the natural climate cycle due to water shortage from the imbalance between evaporation and precipitation, which largely influences human health, agricultural production and ecosystems^1^. As climate is changing with increased temperature and change in precipitation pattern, drought is likely to occur more frequently and its effects are more intense. Especially, losses caused by drought are more severe in developing countries due to the fact that these countries lack resources to cope with drought and climate change^2^. Hence, finding low-cost solutions to mitigate the drought impacts for developing countries is a necessity.

Drought monitoring plays a critical role in drought risk management and is considered the most economically effective drought mitigation measure. In order to serve for drought monitoring, multiple drought indices were developed to quantitatively evaluate drought magnitudes. The American Meteorological Society classified these indices into three groups, namely, meteorological, agricultural, and hydrological droughts, in which the meteorological drought is the most popular for its straightforward interpretation and simple input requirement. The Palmer Drought Severity Index (PDSI)^3^ and the SPI are two widely known meteorological indices^4^. The PDSI incorporates precipitation and temperature data with a simplified water balance model to determine drought conditions. The PDSI is well suited for quantifying long-term drought. However, due to its long memory of the past precipitation, it may not capture the short-term drought. In addition, its lack of spatial consistency makes comparing drought conditions across regions difficult. Compared to the PDSI, the SPI only requires one input (i.e., precipitation), and therefore, it is suitable for the regions where meteorological data are scarce and limited^5^. Another advantage of the SPI is that it can quantify the drought conditions at different time scales (e.g., 1, 3, 6, 9, 12, 24 and 36 months), which allows it to accommodate various water use purposes, such as agricultural irrigation and water supply management^4^. As a result, the SPI is more suitable for representing the drought conditions in developing countries.

Given its popularity, there have been multiple studies using SPI to characterize drought, but with gauged precipitation data^6–9^. However, because the gauge network in many regions of the world is spare and unrepresentative, the SPI derived from the gauged observations does not fully reflect the spatial variations of drought in these regions. With the rapid advancements and availability of remote sensing products in recent years, a wide variety of high-quality global precipitation datasets have been opened for public uses (e.g., Integrated Multi-satellitE Retrievals for Global Precipitation Measurement (IMERG), Tropical Precipitation Measuring Mission (TRMM) Multi-satellite Precipitation Analysis (TMPA), Climate Prediction Centre (CPC) MORPHing technique (CMORPH), Precipitation Estimation from Remotely Sensed Information using Artificial Neural Networks (PERSIANN), Climate Hazards Group InfraRed Precipitation with Stations (CHIRPS), and Multi-Source Weighted-Ensemble Precipitation (MSWEP)). These precipitation datasets are provided at a global scale with a high spatial and temporal resolution, which can be used to monitor drought as an alternative to ground-based ones, especially in the regions where ground-based observations are scarce. Comprehensive reviews on application of different global precipitation products for drought monitoring can be found in^10,11^. 

Among the global precipitation products, the CHIRPS has been widely used for its reliability, long-term availability (1981–present) and fine spatial resolution (0.05°), which is suitable to capture the drought conditions at multiple temporal and spatial scales. The CHIRPS was used to explore the drought conditions in the Lower Mekong Basin and found that the CHIRPS could determine the drought periods and well reflect the spatial variations of drought^12^. The SPI with different time scales computed from the CHIRPS was found to accurately capture drought events and could supplement the ground-based observations for monitoring drought^13^. The comparison results between the CHIRPS with different global precipitation products in Kenya showed that the CHIRPS was the most accurate precipitation products in the tropical warm arid region and could be a reliable data source for drought monitoring^14^. In Iran, the CHIRPS-derived SPI was proven to be suitable for assessing drought at different time scales and climate regions^15^. The CHIRPS was also employed to evaluate drought in the Menderes River basin (Turkey). The results showed that the CHIRPS was highly correlated with the ground-based one and the SPI-3 estimated from this precipitation source can properly monitor the spatial and temporal variability of drought^16^. For its proven usefulness in drought characterization in different regions of the world, the CHIRPS is used in this study to investigate the spatio-temporal variations of drought.

Cuba is an island country situated at the confluence of the northern Caribbean Sea, the Gulf of Mexico and the Atlantic Ocean. In the past three decades, multiple severe drought events have been recorded in Cuba, damaging drinking water resources, increasing forest fire risks, intensifying saline intrusion and affecting crops, livestock and likelihood of local people^17,18^. Cuba’s climate is predicted to be more extreme with increasing average temperature and decreasing annual precipitation by the end of the century^19^, which makes drought effects become increasingly intense. Being the largest agricultural area of Cuba, the Cauto river basin plays a crucial role for supplying food for Cubans but also requires a large volume of water for irrigation, and therefore, is highly vulnerable to drought. Consequently, understanding the spatio-temporal variations of drought in the Cauto river basin is crucial for making drought risk management to mitigate the drought impacts.

Although drought has not been intensively investigated in the Cauto river, there have been several studies on this issue at the national and regional scales. For example, Cuban Institute of Meteorology issues the SPI drought maps every month for the whole country (http://www.insmet.cu). The drought maps are generated by interpolating the SPI calculated from the gauged precipitation data. The Caribbean Drought and Precipitation Monitoring Network implemented an early warning drought system for the Caribbean region. The system uses the gauged precipitation and other auxiliary observations (e.g., water level or soil moisture) to assess the drought conditions and provides the short and seasonal drought predictions using forecasting precipitation^20^. Investigating the reasons for drought in western Cuba found that the modifications in the Caribbean atmospheric circulation, the increased air temperature and the positive anomalies of sea surface temperature were the main factors controlling drought in western Cuba^17^. Drought analysis in Cuba was also performed at different time scales using the SPI, which then was linked to the impacts on water use stakeholders, especially tourism^18^. The characteristics of severe drought events in Cuba were reported and the role of multiple satellite products as useful data sources for on-going drought studies was highlighted^21^. Although the above-mentioned studies provided valuable information for drought monitoring, preparedness and response in Cuba, the spatial variations of drought were not well captured because the drought indices were computed using the gauged observations. In addition, so far there has been no study that thoroughly investigates the drought characteristics and their spatio-temporal variations in Cuba in general and in the Cauto river basin in particular. 

The objective of this study is twofold. First, the study provides a comprehensive scheme to calculate SPI-based drought characteristics from the CHIRPS dataset and evaluates the variations of these characteristics in time and space. Second, the developed scheme is used to explore the spatio-temporal variations of drought in the Cauto River basin using the SPI computed from the CHIRPS that is calibrated by the ground-based precipitation data. The main steps of the study consist of: (1) processing and calibrating the CHIRPS with the ground-based data using Thiessen polygon-based and linear least squares regression methods; (2) calculating the gridded SPI-3, 6, 9 for the Cauto river basin using the CHIRPS; (3) computing drought characteristics, namely, drought frequency, duration, and intensity; (4) evaluating the spatial and temporal variations of drought characteristics in the Cauto river basin using different statistical scores. To the best of our knowledge, this is the first study that develops drought maps from calibrated global precipitation data in the Cauto river. Compared to the current drought maps based on gauged observations in Cuba, these maps have a much higher spatial resolution. This study also provides detailed insights about the drought characteristics including drought intensity, duration and frequency as well as their spatio-temporal variations. It is also worth noting that although the developed scheme in this study is applied for the Cauto river basin with the CHIRPS, it can be effectively applied for other data-scarce regions with other global precipitation data.

## Data availability and methods

### Study area

**Figure 1:**
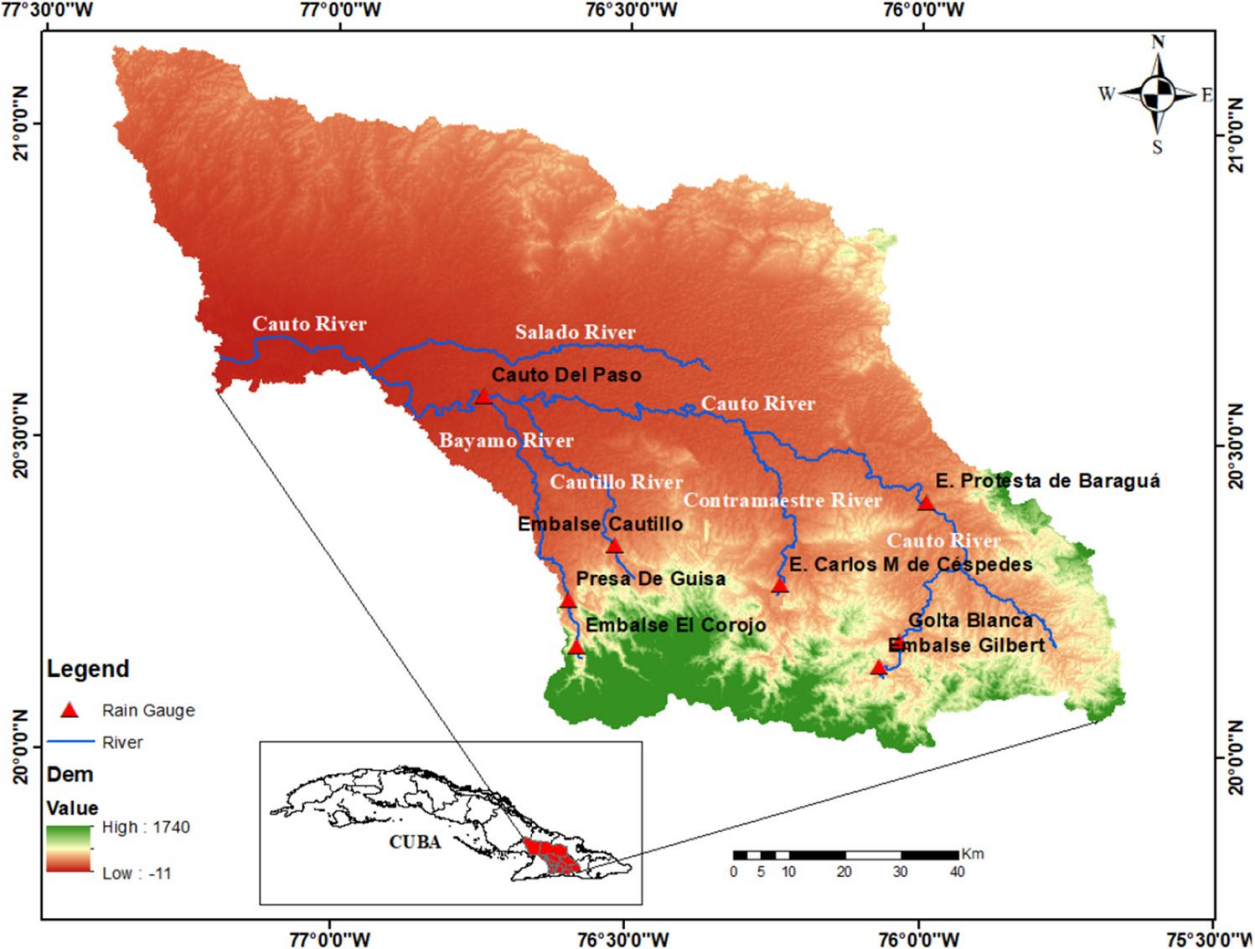
Topographical map and meteorological stations in the Cauto river basin. The map was created with QGIS version 3.16 (https://www.qgis.org).

The Cauto River is the longest river in Cuba with a basin area of 8969 km^2^ and a length of 343 km. The river originates from the foothills of the Sierra Maestra Mountain, at an elevation of 760 m above sea level, and enters the Caribbean Sea (Fig. 1). The main tributaries of the river include Contramaestre (960 km^2^), Bayamo (638 km^2^), Salado (2664 km^2^) and Cautillo (648 km^2^). Characterized by a seasonally humid condition with maritime influence and semi-continental characteristics tropical climate^22^, the river basin has a wet season (May to October) and a dry season (November to April). Of a total annual precipitation of 1206 mm/year, the wet season accounts for 73%. The basin topography is divided into two parts: a mountainous area in the south and southeast with elevations over 300 m and slopes greater than 8°, and a relatively flat northern area suitable for agricultural cultivation (Fig. 1). The Cauto is an important river basin in Cuba where 10% Cuban population lives in four provinces, namely, Granma, Holguin, Santiago de Cuba, and Las Tunas. Agriculture is the main economic activity in the basin, which accounts for 85% of total basin income^22,23^. As for land use, crops are cultivated along the Cauto River valley, whereas evergreen forests cover the upper part of the basin and wetland at the river estuary. Although there are several reservoirs in the river to maintain water supply for irrigation and domestic use, water shortages usually occurs in the river basin during the dry season due to a poor irrigation system.

### Data availability

#### Ground-based precipitation

Ground-based precipitation data were used in this study to evaluate and calibrate the CHIRPS precipitation data. Monthly precipitation data from eight meteorological stations (Fig. 1) during the 2000–2020 period in the Cauto River basin were collected from the Cuban National Institute of Hydraulic Resources. As shown in Fig. 1, these stations are located at the upstream and the middle of the river basin, which are not sufficient to capture the spatial variations of precipitation and drought condition in the entire basin. Table 1 shows the average monthly and annual precipitation of eight stations in the Cauto River basin during the 2000–2020 period. Average monthly precipitation varies from 16 to 112 mm in the dry season and from 89 to 204 mm in the rainy season. Annual precipitation of meteorological stations ranges from 1126 to 1378 mm. The temporal variations of all stations exhibit a similar trend which follows a bimodal pattern with two maxima in May and October, while the minimum precipitation is usually observed in February. This pattern is typical for the Caribbean which is controlled by the moisture convergence originated from the eastern Pacific and Atlantic intertropical convergence zone and the western flank of the North Atlantic subtropical high (NASH) in which the expansion and contraction of the western flank of NASH is the key factor that generates the seasonal variations of precipitation in Cuba and the northwestern Caribbean^24^.

**Table 1:** Monthly and annual precipitation (mm) at eight meteorological stations in the Cauto river basin during the 2000–2020 period. Locations of these stations are shown in Fig. 1.

Station	Month												
	I	II	III	IV	V	VI	VII	VIII	IX	X	XI	XII	Total
Presa De Guisa	55.1	36.2	59.3	101.8	204.0	140.7	132.1	149.7	165.8	187.2	78.4	67.9	1378.2
Cauto del Paso	18.2	17.0	25.9	86.5	174.6	129.8	113.6	124.6	153.0	150.2	45.3	35.2	1073.9
Embalse El Corojo	55.1	36.1	48.9	99.5	188.2	127.7	98.9	121.8	176.6	172.9	85.8	55.6	1267.1
Embalse Cautillo	38.6	25.6	38.7	103.6	189.6	109.8	102.3	133.9	156.8	128.4	55.7	43.4	1126.4
Embalse Carlos M de Céspedes	34.1	24.4	46.5	104.7	172.0	124.8	129.1	131.3	140.9	126.8	72.8	48.0	1155.4
Embalse Gilbert	34.2	20.8	46.8	112.6	163.3	98.5	96.4	142.3	160.6	172.4	89.8	59.2	1196.9
Embalse Protesta de Baraguá	16.3	19.6	32.5	99.1	203.5	147.0	165.2	163.1	142.3	159.9	62.4	34.2	1245.1
Gota Blanca	25.0	19.5	54.2	109.4	169.7	110.0	89.2	139.4	155.2	195.2	84.7	53.7	1205.2

#### CHIRPS precipitation

**Figure 2:**
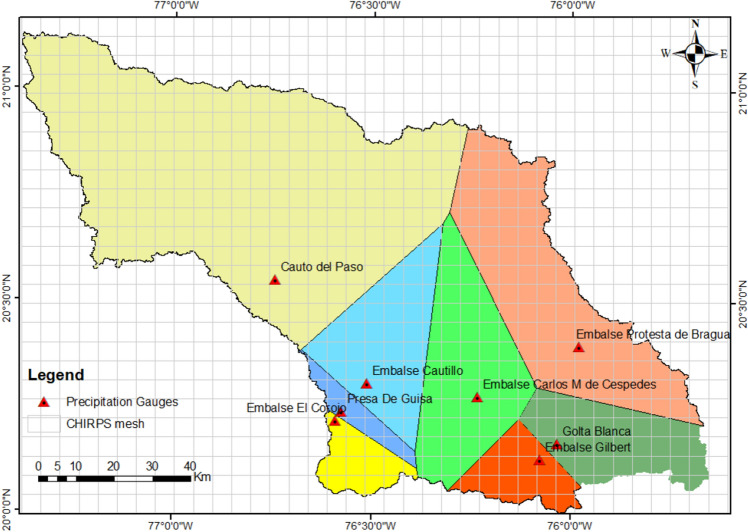
The CHIRPS grid mesh and Thiessen polygons for the Cauto river basin. The map was created with QGIS version 3.16 (https://www.qgis.org).

This study used the CHIRPS precipitation data (version 2.0) to calculate the SPI and evaluate the drought condition in the Cauto River basin. CHIRPS version 2.0 is developed by the Climate Hazards Center (CHC) at UC Santa Barbara (https://data.chc.ucsb.edu/products/CHIRPS-2.0/). CHIRPS combines precipitation from five different satellite products, with more than 2000 ground monitoring stations for calibration. The CHIRPS data features high spatial resolution (~ 5 km) and is updated bi-daily. It encompasses daily, weekly, and monthly records from 1981 to the present, covering a range extending from 50° S to 50° N. This study used the CHIRPS data at the monthly scale during the 1981–2023 period for characterizing drought conditions in the Cauto River basin. In total, the basin contains 434 grid cells (Fig. 2). As shown in Fig. 2, the density of the CHIRPS data is much higher than that of meteorological stations. As a result, it is expected that the spatial variations of drought are better characterized when using the CHIRPS data.

### Methods

**Figure 3:**
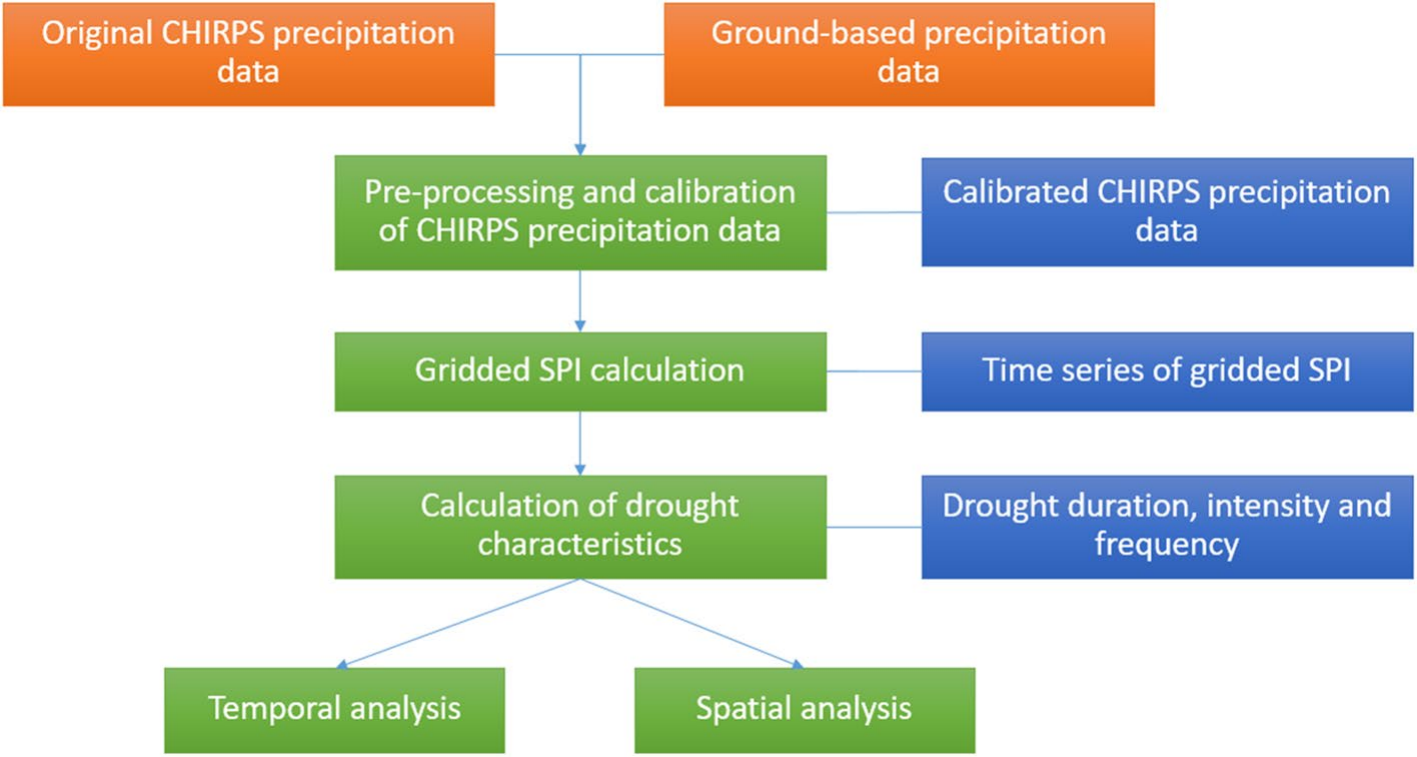
Procedure to perform spatial–temporal analysis of drought condition using the CHIRPS precipitation in the Cauto river basin. The orange box refers to the input variable, while the blue box denotes the output of each step, which is the input of the next step. The green boxes represent the steps to implement pre-processing and data calibration of the CHIRPS and spatial–temporal analysis of drought characteristics.

Procedure that we followed to process and calibrate the CHIRPS precipitation data, to compute the SPI and its characteristics, and to analyze its spatio-temporal variations at the Cauto River basin is illustrated in Fig. 3. This procedure is explained in detail as follows.

#### Pre-processing and calibration of CHIRPS data

Although the original CHIRPS data were globally calibrated by using the ground-based precipitation, this calibration relied on sparse ground-based observations. Hence, it is necessary to locally implement the further calibration of this precipitation data for a specific region. In this study, the ground-based precipitation data obtained from eight meteorological stations in the Cauto river basin were used to calibrate the CHIRPS data. Before calibration, the CHIRPS data at 434 grid cells from 1981 to 2023 within the Cauto River basin were obtained from global datasets. Then, the calibration of CHIRPS data was performed as follows:

*Determination of Thiessen polygons*: Thiessen polygons are defined as the polygons corresponding to a set of points such that any location inside the polygon belonging to a specific point (controlling point) is the nearest to that point. In this study, the Thiessen polygons were determined for the eight meteorological stations in the Cauto River basin using GIS tools. The eight Thiessen polygons corresponding to eight meteorological stations are shown in Fig. 2.

*Calibration of CHIRPS precipitation*: Assuming that the precipitation within each Thiessen polygon exhibits a high correlation with its controlling meteorological station, the CHIRPS precipitation was calibrated using the linear regression equation below:

1 $$P_{{\text{CHIRPS\;}}}^{cali} = a{P_{{\text{CHIRPS}}}} + b$$

In this equation, ${P_{{\text{CHIRPS}}}}$ and $P_{{\text{CHIRPS\;}}}^{cali}$ represent the original and calibrated CHIRPS precipitation respectively; *a* and *b* are coefficients which are determined by the least-squares method as follows:

2 $$a = \frac{{n\mathop \sum \nolimits_{i = 1}^n {P_{CHIRPS,i}}{P_{G,i}} - \mathop \sum \nolimits_{i = 1}^n {P_{CHIRPS,i}}\mathop \sum \nolimits_{i = 1}^n {P_{G,i}}}}{{n\mathop \sum \nolimits_{i = 1}^n P_{CHIRPS,i}^2 - {{\left( {\mathop \sum \nolimits_{i = 1}^n {P_{CHIRPS,i}}} \right)}^2}}}$$

3 $$b = \frac{{\mathop \sum \nolimits_{i = 1}^n {P_G}\mathop \sum \nolimits_{i = 1}^n P_{CHIRPS,i}^2 - \mathop \sum \nolimits_{i = 1}^n {{\bar P}_{CHIRPS,i}}\mathop \sum \nolimits_{i = 1}^n {P_{CHIRPS,i}}{P_{G,i}}}}{{n\mathop \sum \nolimits_{i = 1}^n P_{CHIRPS,i}^2 - {{\left( {\mathop \sum \nolimits_{i = 1}^n {P_{CHIRPS,i}}} \right)}^2}}}$$

in which ${P_{G,i}}$ is the ground-based precipitation at the meteorological station *G*th at time *i*th; $P_{\text{CHIRPS,i}}$ is the average CHIRPS precipitation of all grid cells inside the Thiessen polygon corresponding to the station *G*th at time *i*th; *n* is the length of precipitation data, which is the total number of monthly precipitation data from 2000 to 2020. For each Thiessen polygon, a regression equation was formulated. In total, eight linear regression equations were developed for the Cauto River basin.

In order to compare the original and calibrated CHIRPS precipitation with the ground-based one, the following criteria were used:

4 $$NSE = 1 - \frac{{\mathop \sum \nolimits_{i = 1}^N {{\left( {{P_{G,i}} - {P_{CHIRPS,i}}} \right)}^2}}}{{\mathop \sum \nolimits_{i = 1}^N {{\left( {{P_{G,i}} - \overline {P_G} } \right)}^2}}}$$

5 $$r = \frac{{\mathop \sum \nolimits_{i = 1}^N \left( {{P_{G,i}} - \overline {P_G} } \right)\left( {{P_{CHIRPS,i}} - \overline {{P_{CHIRPS}}} } \right)}}{{\sqrt {\mathop \sum \nolimits_{i = 1}^N {{\left( {{P_{G,i}} - \overline {P_G} } \right)}^2}} \sqrt {\mathop \sum \nolimits_{i = 1}^N {{\left( {{P_{CHIRPS,i}} - \overline {{P_{CHIRPS}}} } \right)}^2}} }}$$

6 $$BIAS = \frac{{\mathop \sum \nolimits_{i = 1}^N {P_{CHIRPS,i}} - \mathop \sum \nolimits_{i = 1}^N {P_{G,i}}}}{{\mathop \sum \nolimits_{i = 1}^N {P_{G,i}}}} \times 100\%$$

In which *NSE*, *r,* and *BIAS* are the Nash–Sutcliffe, Pearson correlation, and bias criteria, respectively. The agreement between these two types of precipitation is good if the $NSE$ and r approach one and the *BIAS* is near zero.

#### Computation of gridded SPI

SPI is based on the probability of precipitation over any duration of interest that gives a sample of abnormal wetness and dryness. The frequency distribution of precipitation is well described by a two-parameter Gamma probability density function which is formulated by:

7 $$G(x) = \frac{1}{\Gamma \left( \alpha \right)}\mathop \smallint \limits_0^x {t^{\alpha - 1}}{e^{ - t}}dt\;$$

In which $\Gamma \left( \alpha \right)$ is the gamma function; $\alpha$ and $\beta$ are the shape and scale parameters of the gamma distribution that can be estimated using the maximum likelihood approach; *x* denotes precipitation accumulation.

Instead of using the Gamma distribution function, an empirical non-parametric probability method was proposed^25^. Accordingly, the marginal probability of precipitation is firstly calculated using the empirical Gringorten plot location:

8 $$p\left( {x_i} \right) = \frac{i - 0.44}{{n + 0.12}}$$

In which *n* is the sample size, *i* is the rank of precipitation data in a series sorted from the smallest to largest, *p*(*x*_*i*_) is the corresponding empirical probability. Next, the empirical probability $p\left( x \right)$ is transformed to the *SPI* as:

9 $$SPI = {\emptyset^{ - 1}}\left( {p\left( x \right)} \right)$$

In which $\emptyset$ represents the inverse standard normal distribution function with the mean of zero and standard deviation of one. Because there is no analytical solution to Eq. (9), $SPI$ can be approximately computed as:

10 $$SPI = \left\{ {\begin{array}{*{20}{c}} { - \left( {t - \frac{{{C_0} + {C_1}t + {C_2}{t^2}}}{{1 + {d_1}t + {d_2}{t^2} + d{t^3}}}} \right),}&{\quad 0 < p \leq 0.5} \\ { + \left( {t - \frac{{{C_0} + {C_1}t + {C_2}{t^2}}}{{1 + {d_1}t + {d_2}{t^2} + d{t^3}}}} \right),}&{\quad 0.5 < p \leq 1} \end{array}} \right.$$

In which *C*_*0*_ = 2.515517; *C*_*1*_ = 0.802583; *C*_*2*_ = 0.010328; *d*_*1*_ = 1.432788; *d*_*2*_ = 0.189269; *d*_*3*_ = 0.001308; and $$t = \left\{ {\begin{array}{*{20}{c}} {\sqrt {ln\frac{1}{{p^2}}} ,}&{\quad 0 < p \leq 0.5} \\ {\sqrt {ln\frac{1}{{{{\left( {1 - p} \right)}^2}}}} ,\;}&{\quad 0.5 < p \leq 1} \end{array}} \right.$$.

Based on the SPI calculated by Eq. (10), the drought severity can be classified into seven levels from extremely wet to extremely dry conditions as shown in Table 2^26^. The table shows that drought occurs when SPI is below − 1.0. Data used for SPI calculation should have a minimum length of 30 years to address the cycle of climate variability. In this study, the SPI was calculated using the calibrated CHIRPS data with a length 43 years (1981–2023). The SPI was calculated for each grid cell to generate the time series of gridded SPI which then was used for evaluating the spatio-temporal variations of drought.

**Table 2:** Classification of drought magnitude.

No	SPI values	Classification
1	$SPI > 2.0$	Extreme wet
2	$1.5 < SPI \leq 2.0$	Severe wet
3	$1.5 < SPI \leq 1.0$	Moderate wet
4	$- 1.0 < SPI \leq 1.0$	Normal
5	$- 1.5 < SPI \leq - 1.0$	Moderate drought
6	$- 2.0 < SPI \leq - 1.5$	Severe drought
7	$SPI \leq - 2.0$	Extreme dry

#### Calculation of drought characteristics

In addition to drought magnitude determined in Table 2, the drought condition was also typically characterized by its frequency, duration, and intensity^27^. Drought frequency measures how often drought occurs and is computed as the ratio of number of drought months to the total number of months in dataset in which drought month is defined as the month with a SPI below − 1. Drought duration (*D*) and intensity (*I*) refer to the length and the strength of drought in a period. A drought event begins when SPI is below − 1 and ends as it is greater or equal to zero^27^. Drought duration and intensity of a drought event are the total number of months and the total of absolute SPI values in that event. Drought duration and intensity are expressed as the average of durations and intensities of drought events as follows:

11 $$D = \frac{1}{M}\mathop \sum \limits_{m = 1}^M {d_m}$$

12 $$I = \frac{1}{M}\mathop \sum \limits_{m = 1}^M {i_m}$$

in which *M* is the number of drought events; ${d_m}$ and ${i_m}$ are the duration and intensity of drought event *m*th*.*

#### Spatio-temporal analysis of drought

In order to analyze the long-term temporal trend of drought condition, we used the Mann–Kendall (MK) test, which specifies if a time series is monotonically upward or downward by measuring differences in signs between earlier and later data points organized in chronological order. The advantage of the MK test is that it is a non-parametric test, and therefore, does not require assumption of distribution of data. The null hypothesis for this test is that the time series have no trend. In the MK Test, the MK test statistic is calculated as follows:

13 $${Z_{MK}} = \left\{ {\begin{array}{*{20}{c}} {\frac{S - 1}{{\sqrt {VarS} }},\;}&{\quad S > 0} \\ {0,\;}&{\quad S = 0} \\ {\frac{S + 1}{{\sqrt {VarS} }},}&{\quad S < 0} \end{array}} \right.$$

in which *S* and its variance *VarS* are defined as follows:

14 $$S = \mathop \sum \limits_{i = 1}^{n - 1} \mathop \sum \limits_{j = i + 1}^n sgn\left( {{x_j} - {x_i}} \right)$$

15 $$VarS = \frac{1}{18}\left[ {n\left( {n - 1} \right)\left( {2n + 5} \right) - \mathop \sum \limits_{t = 1}^G {f_t}\left( {{f_t} - 1} \right)\left( {2{f_t} + 5} \right)} \right]$$

where *x*_*i*_ is a member *i*th of time series; *n* is the length of time series; *g* is the number of tied groups and *f*_*t*_ is the number of values in tied group *t*; $sgn$ is the signum function.

Testing trends is performed for a specific α significance level. The null hypothesis is rejected and time series exhibits a trend if $\left| Z \right| > {Z_{1 - \frac{\alpha }{2}}}$ in which the trend is upward if Z is positive and downward if *Z* is negative. In this study the significance level was set at 5%, corresponding with ${Z_{1 - \frac{\alpha \;}{2}}}$ = 1.96.

In order to determine the homogeneity of drought characteristics in space, we employed the coefficient of variation (*C*_*v*_) which is specified by the ratio between the standard deviation and the mean. The higher *C*_*v*_ indicates that the degree of spatial homogeneity is low and vice versa.

## Results

### Calibration of CHIRPS precipitation

**Figure 4:**
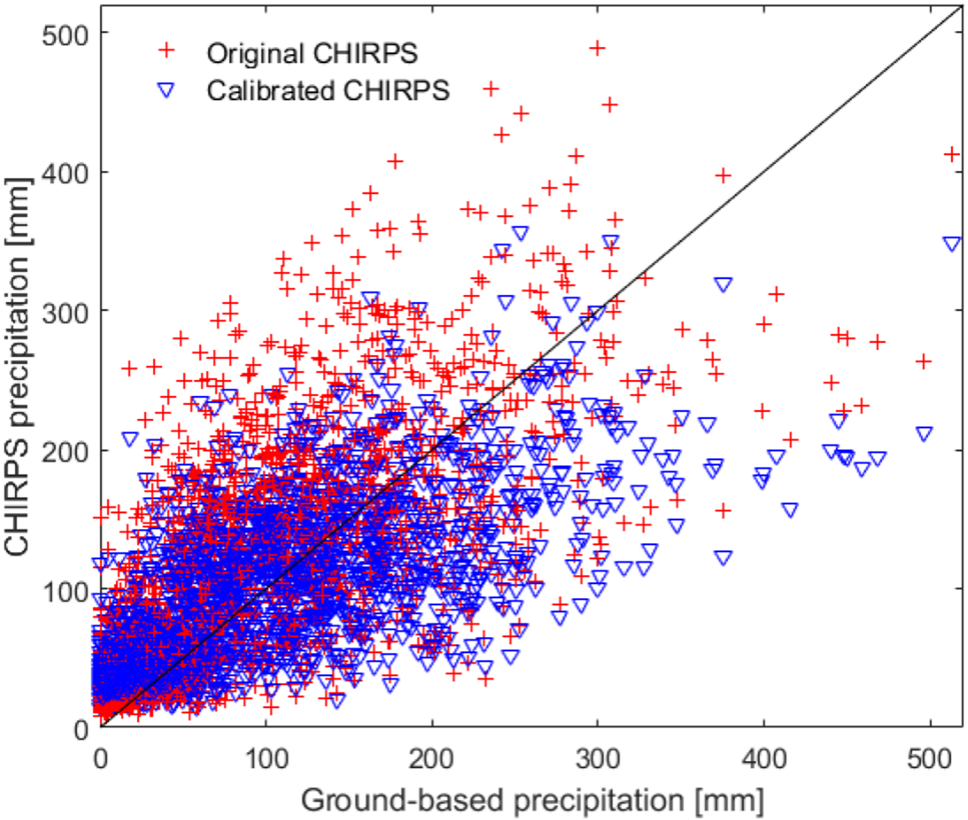
Comparison of monthly ground-based precipitation and CHIRPS at eight meteorological stations in the Cauto river basin.

Comparison between the ground-based precipitation obtained from eight meteorological stations and the CHIRPS precipitation is presented in Fig. 4 and Table 3. Because the ground-based precipitation was only available for the 2000–2020 period, we used data from this period for comparison. The CHIRPS corresponding to a given meteorological station was obtained by averaging the CHIRPS at all grid cells within the Thiessen polygon controlled by that station. Quantitative evaluation of the agreement between the two precipitation sources was performed using the correlation coefficient, *BIAS* and *NSE*. The results showed that the two precipitation sources correlated well with each other with a correlation coefficient ranging from 0.65 to 0.74, in which the highest correlation was found at the Gonta Blanca station and the lowest correlation was observed at the Embalse Carlos M de Cespedes and Presa De Guisa stations. However, the CHIRPS precipitation was considerably higher than ground-based one. Except for the Embalse Protesta de Bragua station (*bias* = 0.03, *NSE* = 0.49), the bias was quite high (from 0.23 to 0.46) and the *NSE* was relatively low (− 0.14 to 0.49). Especially, at the Embalse El Corojo station, the *NSE* was below zero and the bias was equal to 0.46, indicating that the discrepancy between the CHIRPS and ground-based precipitation was significantly large. The main reason for this disagreement may come from the difference in the scales of two observations. While the CHIRPS precipitation was averaged over the Thiessen polygon, the meteorological station locally observed the precipitation surrounding it. The measurement errors of both CHIRPS and ground-based precipitation also contributed to this disagreement. Further observations revealed that the agreement between the ground-based and CHIRPS precipitation as better when the precipitation was lower than 200 mm. This can be explained by the fact that it is more challenging to accurately estimate high precipitation from satellite observations. In addition, the difference between the two sources at the southwest of the river basin (at Presa De Guisa and Embalse El Corojo stations) was higher than that at the southeast (at Embalse Protesta de Bragua, Golta Blanca and Embalse Gilbert stations). This was because the southwest part of the river basin is dominated by forest and steep slope topography which makes the CHIRPS observation less accurate.

**Table 3:** Comparison criteria between the measured precipitation and CHIRPS before and after calibration at eight meteorological stations in the Cauto river.

No	Meteorological station	Original CHIPS			Adjusted CHIPS		
		*r*	*BIAS*	*NSE*	*r*	*BIAS*	*NSE*
1	Embalse Carlos M de Cespedes	0.65	0.25	0.17	0.65	0	0.42
2	Embalse Gilbert	0.71	0.23	0.42	0.71	0	0.51
3	Embalse Protesta de Bragua	0.72	0.03	0.49	0.72	0	0.52
4	Golta Blanca	0.74	0.27	0.42	0.74	0	0.54
5	Presa De Guisa	0.65	0.27	0.10	0.65	0	0.42
6	Cauto del Paso	0.69	0.03	0.44	0.69	0	0.48
7	Embalse El Corojo	0.70	0.46	− 0.14	0.70	0	0.49
8	Embalse Cautillo	0.70	0.23	0.31	0.70	0	0.49

**Figure 5:**
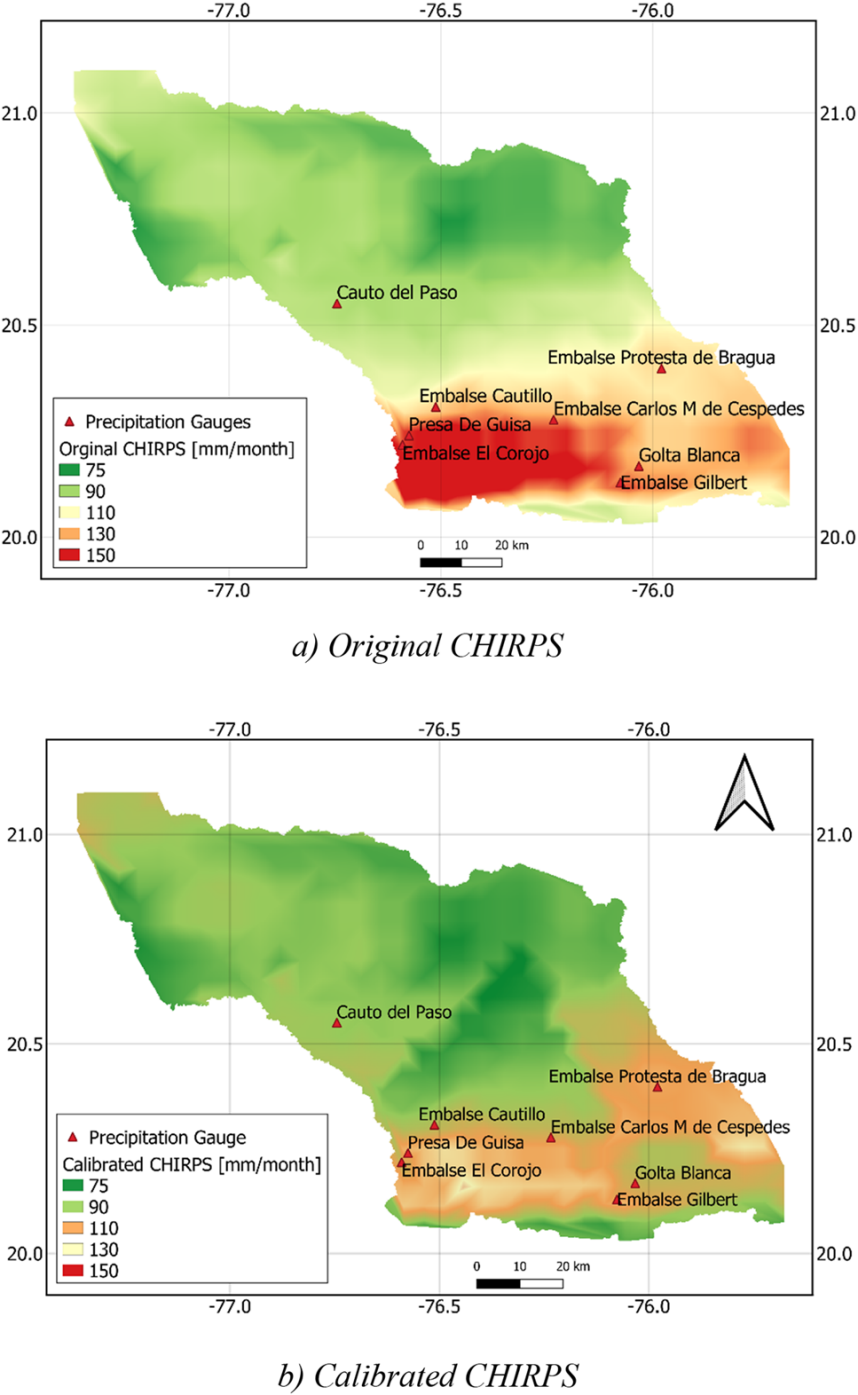
Comparison of temporally-averaged monthly CHIRPS before (**a**) and after calibration (**b**). The map was created with QGIS version 3.16 (https://www.qgis.org).

In order to improve the accuracy of the original CHIRPS precipitation, calibration of this precipitation data was performed. Linear calibration equations were constructed between the CHIRPS and ground-based precipitation from historical data. Then, these equations were used to calibrate the CHIRPS. Figure 5 compares the original and calibrated CHIRPS precipitation with the ground-based one at the eight meteorological stations and Table 3 compares the BIAS, correlation coefficient, and *NSE* criteria before and after calibration. The obtained results showed that the calibrated CHIRPS agreed better with the ground-based observation than the original one. Because the linear calibration equations were obtained by the least-square method, the bias equaled to zero and the correlation coefficient remained unchanged after the CHIRPS was calibrated. The *NSE* significantly increased at all meteorological stations, ranging from 0.42 to 0.54. The maximum improvement was observed at the Embalse El Corojos station where the *NSE* increased from − 0.14 to 0.49. This suggested that the calibration helped to reduce the difference between the ground-based and CHIRPS precipitation, and therefore, the eight calibration equations for the eight Thiessen polygons could be effectively employed to calibrate the CHIRPS precipitation.

Figure 5 compares the temporally-averaged CHIRPS maps in the Cauto River basin before and after calibration. The figure shows that the CHIRPS with its high spatial resolution much better captured the spatial pattern of precipitation than the ground-based one. In addition, the spatial distribution of precipitation shown by the two maps was approximately similar in which the precipitation at the upper region of the river basin was much higher than the lower region due to the cooling of air with increasing altitude. However, after calibration, the differences in precipitation between the two regions were noticeably reduced. Figure 5 shows that while the monthly precipitation corresponding to the original CHIRPS ranged from 75 to 180 mm/month, this range was 75–130 mm/month for the calibrated CHIRPS.

**Figure 6:**
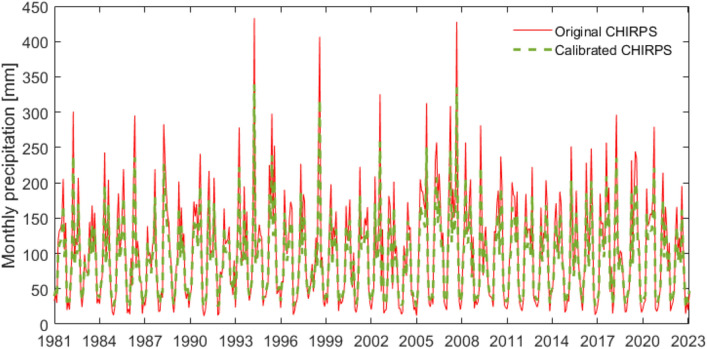
Monthly MAP calculated from the original and calibrated CHIRPS.

Figure 6 compares the mean area precipitation (MAP) of the original and calibrated CHIRPS precipitation. The figure indicates that there was generally a good agreement between the original and calibrated CHIRPS. The maximum of the original CHIRPS precipitation was higher than that of the calibrated one. Conversely, the minimum of the original CHRPS precipitation was lower. This can be explained by the fact that compared to the ground-based observations, the CHIRPS underestimated low precipitation and overestimated the high precipitation. By calibration with the ground-based observations, difference between lower and higher CHIRPS precipitation reduced.

### Characterization of drought variability

#### Calculation of SPI drought index

In order to analyze the spatio-temporal variations of the drought condition in the Cauto River basin, the study calculated the SPI for 434 CHIRPS grid cells within the river basin. First, the monthly CHIRPS at 434 grid cells during the 1981–2023 period was calibrated using the calibration equations developed in the previous step. Next, the monthly MAP for the whole Cauto River basin was calculated by averaging the monthly calibrated CHIRPS precipitation at all 434 grid cells. Finally, the MAP and calibrated CHIRPS at each grid cell were summed up at different time scales (3, 6 and 9 months) to calculate the SPI-3, 6, 9, respectively.

#### Temporal characterization of drought

**Figure 7:**
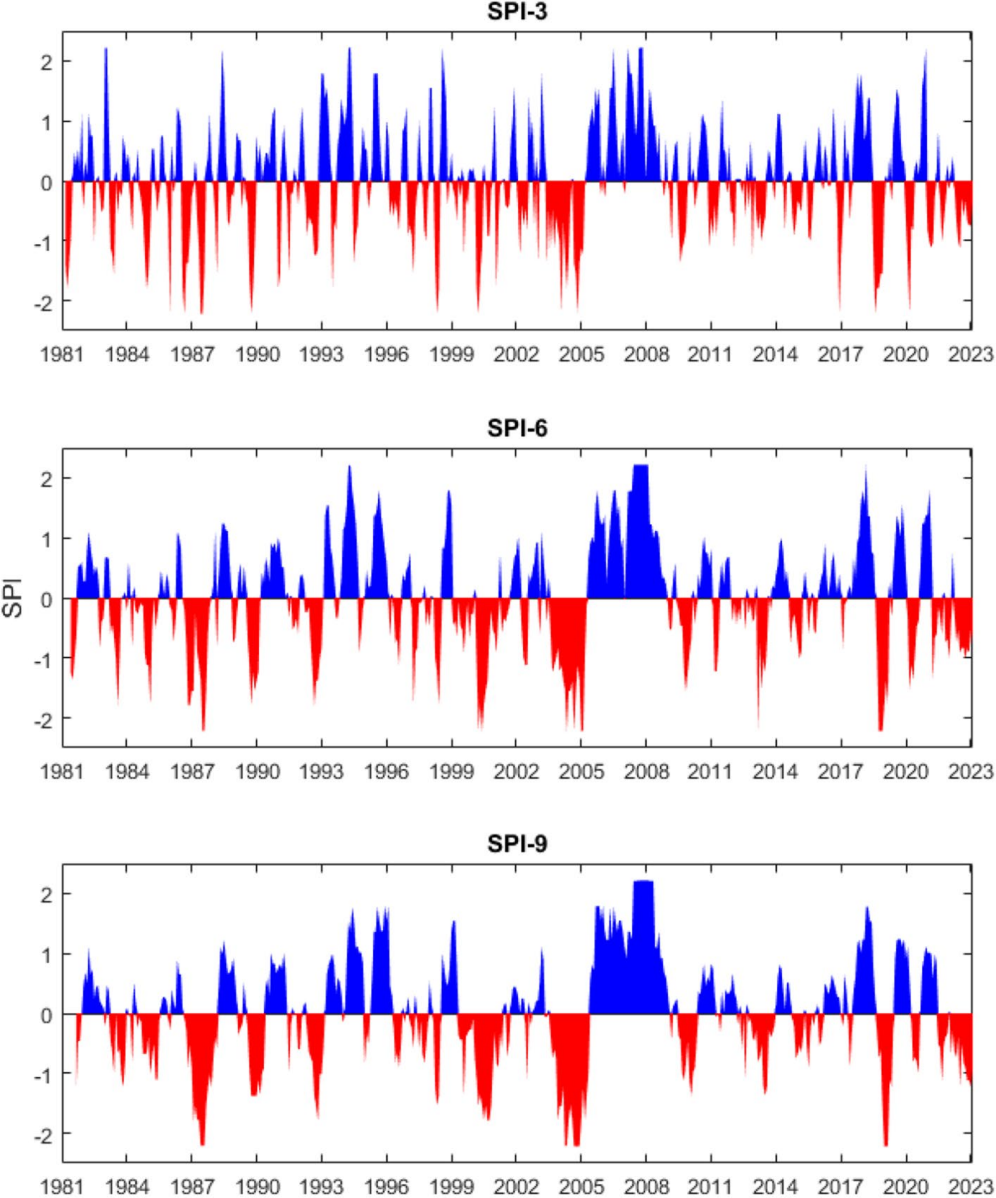
Temporal variations of SPI-3, 6, 9 in the Cauto River basin in the 1981–2023 period. The SPI index was calculated from the CHIRPS-derived MAP of Cauto River basin.

The temporal characterization of the drought conditions was analyzed using the SPI with time scales of 3, 6, 9 months computed from the CHIRPS-derived MAP of the Cauto River basin. Figure 7 presents the temporal trends of SPI during the 1981–2023 period (43 years). Thanks to the CHIRPS data, the long-term variations in drought were investigated. During the 43-year period, multiple drought events were observed in the Cauto River basin. The number of drought months (SPI < − 1) were 84, 81, and 78, which corresponds to drought frequencies of 16.6%, 16.0%, and 15.4% at the time scales of 3, 6, 9 months, respectively (Table 4). The drought duration increased from 3.8 to 7.6 months and the drought intensity increased from 4.1 to 8.8 when the time scale changed from 3 to 9 months. The driest years (SPI < − 1.5) were identified as 1987–1988, 1990, 1992–1993, 1998, 2000–2001, 2004–2005, and 2019 in which the 2004–2005 period was considered as the worst drought event in the Cauto River basin in the 1981–2023 period. After the 2004–2005 drought period, we observed the wettest and longest period lasting from 2006 to 2009. These findings are similar to the drought data documented in^21^, indicating that the SPI was capable to well capture the occurrence time, frequency, duration, and intensity of drought events. There are multiple factors that control the appearance of drought events. At regional scale, tropical Pacific and North Atlantic oceans govern the drought variability, whereas at a local scale, the topography might play important role in the drought variations^27^. Other study suggested that the drought events occurred in the April–November period were closely linked with the peak season Atlantic Meridional Mode (AMM), while in the July–November period, drought was strongly related to North Atlantic Oscillation (NAO). If AMM, NAO and western Pacific ENSO are assumed to concurrently occur, ENSO exhibits the strongest impact, followed by AMM and NAO^28^.

**Table 4:** Number of drought events corresponding to different time scales and levels of drought condition during the 1981–2023 period.

Drought characteristics	SPI-3	SPI-6	SPI-9
Drought magnitude [months]			
Moderate drought	48	45	42
Severe drought	24	24	24
Extreme drought	12	12	12
Drought frequency [%]	16.6	16.0	15.4
Drought duration [months]	3.8	6.0	7.6
Drought intensity [–]	4.1	6.9	8.8

Figure 7 and Table 4 also show the impact of the time scale (3, 6 and 9 months) on the SPI. All severe and extreme drought events are well observed by the SPI-3, 6, 9. The occurrence time of drought events was approximately the same at all time scales. The number of severe and extreme drought ($SPI \leq - 1.5$) months were identical for all time scales. However, for the moderate drought ($- 1.5 < SPI \leq - 1.0$), there were 48 drought months observed at the SPI-3 time series, while this number was 45 and 42 months at the SPI-6 and SPI-9 time series. In addition, while the drought frequency decreased with increasing time scale, the drought duration and intensity increased. This indicated that the SPI with a shorter time scale better detected the small and short dry events than the SPI with longer time scale. As a result, the selection of the drought time scale depends on the purpose of the study. If one would see short and small drought events, the short time scale SPI is preferred. Otherwise, long and large drought events are better observed in the SPI with a longer time scale.

The long-term trend of the SPI for each month was quantitatively evaluated using the MK test (Table 5). The MK test statistic ${Z_{MK}}$ value ranged from − 1.73 to + 0.89, − 0.86 to + 0.87, and − 0.28 to + 1.02 for the SPI-3, 6, and 9. These MK test values were lower than 1.96, which corresponds with a significance level of 5%. This implied that there was no significant trend in the long-term drought. However, it is worth noting that there were two opposite directions in the MK test results. ${Z_{MK}}$ tended to be positive in the wet season and negative in the dry season, which signified that although the trends were not very clear, the drought condition in the dry season tended to be more severe, while the wet season was wetter in the 1981–2023 period.

**Table 5:** Results of MK test for the SPI with time scales of 3, 6 and 9 months during the 1981–2023 period.

Month	I	II	III	IV	V	VI	VII	VIII	IX	X	XI	XII
SPI-3	− 0.22	− 0.91	− 1.73	− 0.35	0.48	0.22	0.24	0.35	0.61	0.91	0.76	0.54
SPI-6	0.87	0.30	− 0.43	− 0.57	− 0.10	− 0.86	0.02	0.46	0.78	0.78	0.82	0.67
SPI-9	0.76	0.50	0.02	0.75	0.46	− 0.03	− 0.28	− 0.24	− 0.01	0.63	0.78	1.02

#### Spatial characterization of drought

**Figure 8:**
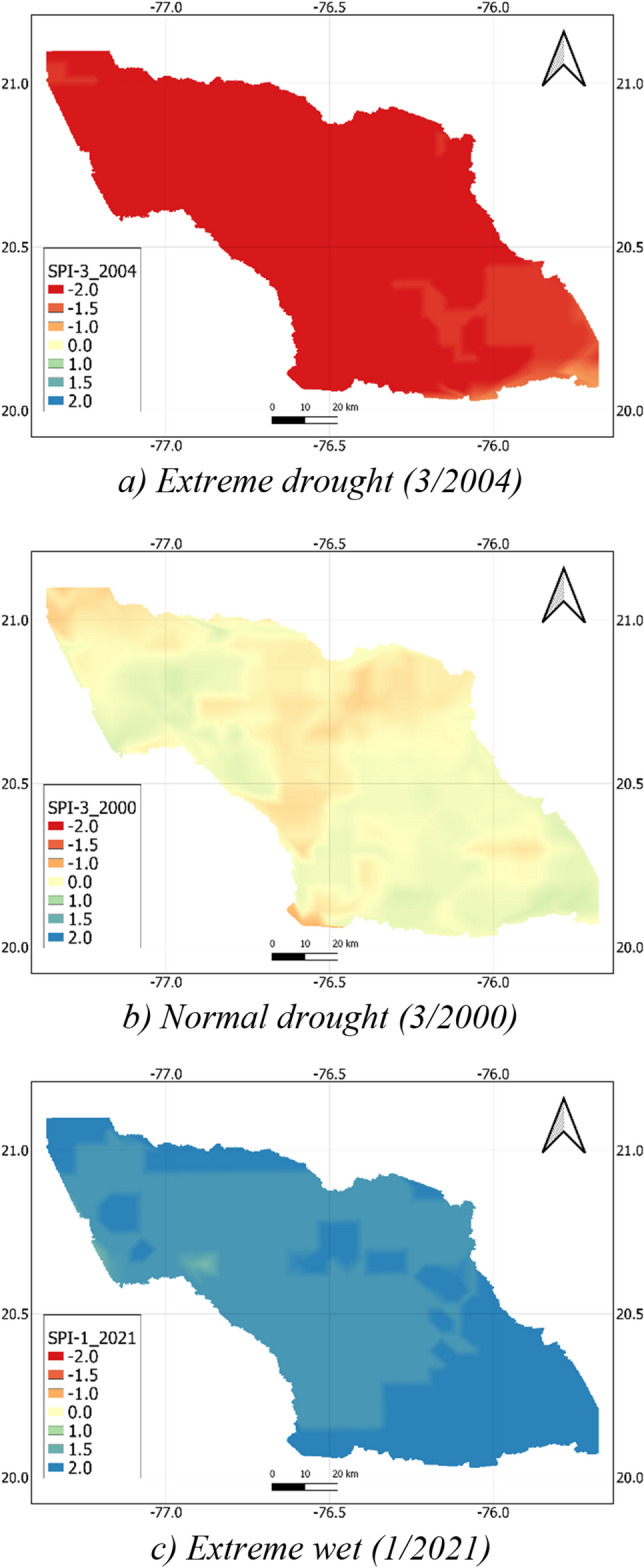
Examples of SPI-3 drought map of Cauto River basin for (**a**) dry, (**b**) normal and (**c**) wet month. The map was created with QGIS version 3.16 (https://www.qgis.org).

One of the advantages of the CHIRPS for quantifying the drought conditions is that it provides drought information with a higher spatial resolution than other global precipitation datasets. Figure 8 shows the SPI-3 maps obtained by the calibrated CHIRPS precipitation at three levels, namely, extremely wet, normal, and extremely dry conditions for illustration. A similar trend was also observed with other time scales and drought conditions. Generally, the figure points out that the drought condition was relatively spatially homogeneous with a coefficient of variation below 0.12. This indicated that although there were relatively large differences in the precipitation distribution in space (see Fig. 5), the occurrence time of drought was comparatively similar in the whole Cauto River basin. The more homogeneity was seen in the two extreme conditions. The coefficients of variation were 0.11 and 0.1 for the extremely wet and dry conditions, while this value for the normal condition was 0.12. This implies that if extreme (dry/wet) events occur, it is more likely it will occur at the whole river basin.

**Figure 9:**
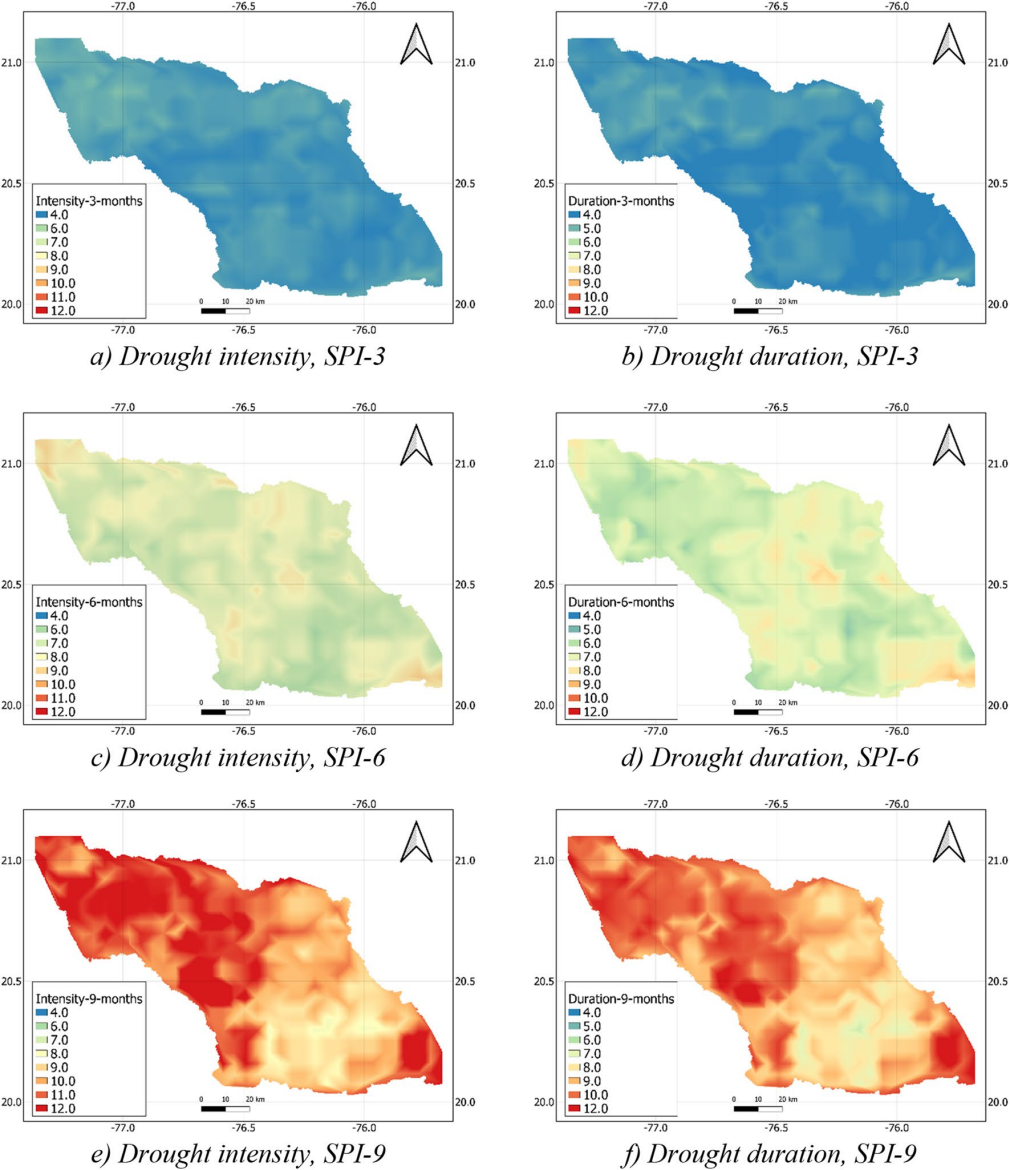
Maps of drought intensity (**a**,**c**,**e**) and duration (**b**,**d**,**f**) in the Cauto River basin corresponding with the SPI with time scales of 3, 6 and 9 months. The map was created with QGIS version 3.16 (https://www.qgis.org).

Figure 9 presents the maps of the drought intensity and duration in the Cauto River basin, calculated from the SPI with the time scales of 3, 6, and 9 months. The drought frequency was not shown here because the spatial distribution of the drought frequency was uniform. The figure indicates that both drought duration and intensity exhibited the upward trend with longer time scale. When the time scale rose from 3 to 9 months, the drought duration increased from 3.6–5.0 to 6.4–13.2 months and the drought intensity increased from 3.9–5.4 to 7.7–14.9. The spatial variations also depended on the time scale. The coefficients of variation of the drought intensity were 0.06, 0.08 and 0.14 at time scales of 3, 6, and 9 months, while these values of the drought duration were 0.06, 0.09, and 0.14. This implies that the spatial variations of drought characteristics exhibited more diverse in the longer time scale. This finding is reasonable because the spatial pattern of precipitation is larger when time scale increases.

It is worth noting that topography is an important factor influencing the spatial variations of drought. Figure 8 shows that the SPI at the south and southeast of the river basin where the topography is characterized by mountains was higher than that at the lower one even in the extremely dry condition. Figure 9 also indicates that, the drought intensity the higher topography was lower, and the drought duration was shorter than the lower altitude area in the south. In brief, although drought concurrently occurred at the entire Cauto river basin, the drought magnitude at the high-altitude area was less intensive than that at the low-altitude area, which is more or less similar to that of precipitation (see Fig. 5).

## Conclusion

This study presented the application of CHIRPS precipitation to characterize the drought conditions in the Cauto River basin, the most important river basin for food and water supply in Cuba. Although it is the largest and the most important river basin in Cuba, so far there have been no studies that intensively analyze the spatio-temporal variations of drought characteristics in the Cauto River. Due to the sparse density of precipitation stations in the Cauto River basin, it is not feasible to analyze the spatio-temporal variations of drought using the gauged precipitation data. In that context, the CHIRPS with a high spatial resolution (0.05°) and long-term availability (1981–present) is a good alternative for characterization of the drought conditions. Comparison results showed that the monthly CHIRPS was highly correlated with the ground-based observations, but the bias between the two precipitation sources was large. In order to further improve the accuracy of the CHIRPS, the study employed the Thiessen polygon-based method and least squares linear regression equations to calibrate the CHIRPS with the ground-based one. After calibration, the differences between the ground-based precipitation and CHIRPS were significantly reduced, and the calibrated CHIRPS could be reliably used for analyzing the spatio-temporal variations of the drought characteristics.

Using the calibrated monthly CHIRPS, the study computed the SPI-3, 6, 9 for the entire the Cauto River basin and investigated the variations of drought characteristics. The results suggested that the SPI was capable of capturing the occurrence time, duration, intensity, and frequency of drought events. When the time scale increased, the drought frequency decreased, while the duration and intensity increased. This signified that the SPI with a short time scale better captured the short and small drought events than the SPI with a long-time scale. The temporal trend analysis by the MK test showed that although there was no significant trend of drought in long-term, drought tended to be more severe in the dry season, while the wet season seemed to be wetter. The spatial analysis revealed that the drought condition was relatively temporally homogeneous, which implied that drought approximately occurred in all locations of the Cauto River basin at the same time. However, the magnitude of drought at the high altitude is slightly smaller than that at the low altitude.

## Discussion

This study is an example of how the global precipitation datasets can be combined with the gauged precipitation for drought characterization in data-scarce regions. We showed that the CHIRPS precipitation after calibration with few gauge observations was very useful for monitoring drought events and assessing the spatial–temporal variations of drought in the Cauto River basin, which is crucial for developing drought management plans to mitigate damages caused by drought for the river basin. Methods developed for the Cauto River basin using the CHIRPS precipitation in this study can be well applied for other regions with other global precipitation data sources. Especially, with increasing availability of the near real-time global precipitation data in recent years, it is feasible to develop a low-cost drought monitoring and early warning system based on these data. This approach is particularly useful in developing countries such as Cuba, where limited financial resources result in a scarcity of real-time, ground-based observations that fail to meet the demands for effective drought monitoring.

Although important results were obtained in this study, several shortcomings remain to be addressed in future research. Besides precipitation, other hydro-meteorological variables such as air temperature, evapotranspiration, and soil moisture may influence the drought conditions. However, due to the lack of these data, our study used the SPI, which is computed solely from precipitation to characterize drought. As a result, the SPI may not fully represent the drought conditions as well as its spatial–temporal variations in the Cauto River basin. Moreover, because the SPI does not consider evapotranspiration, it does not fully address the effects of temperature, and consequently, climate change on drought. Therefore, it would be interesting to compare the SPI with other drought indices in future studies. In addition, the CHIRPS precipitation data were selected to calculate the SPI and characterize the drought conditions for its high spatial resolution and long-term availability. However, several other global precipitation datasets (e.g., IMERG, TRMM, TMPA, CMORPH, PERSIANN, MSWEP, etc.) that may provide good quality were not considered in this study. Several studies have also shown that combing multi-source global precipitation datasets with auxiliary data (e.g., gauge precipitation, topography and soil moisture) could improve the accuracy of precipitation estimate^29,30^. Therefore, future studies will quantitatively compare different precipitation products to select suitable datasets for the study area or to merge them together to enhance the quality of precipitation data.

## Data Availability

Data and codes that support the findings of this study are available at https://www.dropbox.com/scl/fo/sd33gma5uahkrbhp351hy/ABBp4BK3_InJIdUGS9DKJWo?rlkey=0jqee1sua3108t2yflpz5nkiw&dl=0.
